# Supplementary material for: Molecular Dynamics Simulation Reveals the Mechanism of Substrate Recognition by Lignin-Degrading Enzymes
Source: Int J Mol Sci. 2025 Sep 25;26(19):9378. doi: 10.3390/ijms26199378 (PMC12525479; doi:10.3390/ijms26199378)
Supplement: Supplementary file 1 [file ijms-26-09378-s001.zip › ijms-3828157-supplementary.pdf]

Supporting Information

Molecular Dynamics Simulation Reveals the Mechanism of Substrate Recognition by Lignin-Degrading Enzymes

Xue Ma [1,†], Xueting Cao [1,†], Zhenyu Ma [1], Jingyi Zhu [2], Letian Yang [1], Min Xiao [1,2,*] and Xukai Jiang [1,*]

[1] National Glycoengineering Research Center, Shandong University, Qingdao 266237, China
[2] State Key Laboratory of Microbial Technology, Shandong University, Qingdao 266237, China
* Correspondence: minxiao@sdu.edu.cn (M.X.); xukai.jiang@sdu.edu.cn (X.J.)
† These authors contributed equally to this work

This file contains:

Figure S1. Time evolution of backbone RMSD in lignin-degrading enzymes with and without substrate.

Figure S2. Interaction energy between substrate and lignin-degrading enzyme.

Figure S3. Binding Free energy between lignin-degrading enzymes and substrates.

Figure S4. Hydrogen bonding interactions between substrate and lignin-degrading enzymes.

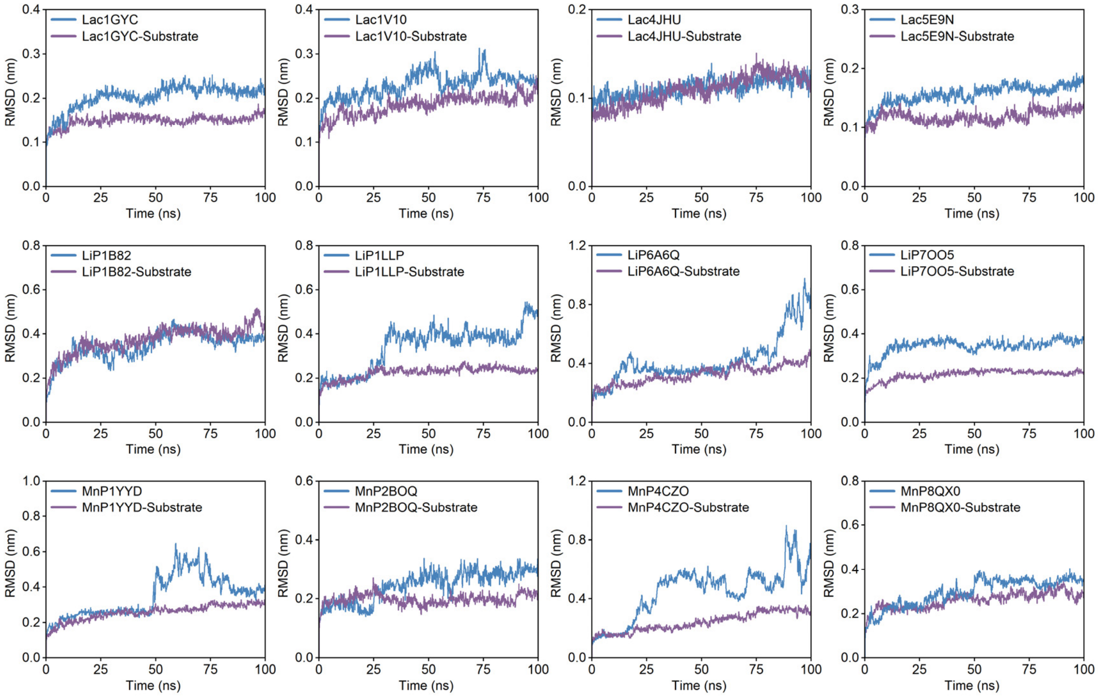

Figure S1. Time evolution of backbone RMSD in lignin-degrading enzymes with and without substrate. Backbone RMSD fluctions are shown for the substrate-free (blue) and substrate-bound (purple) states.

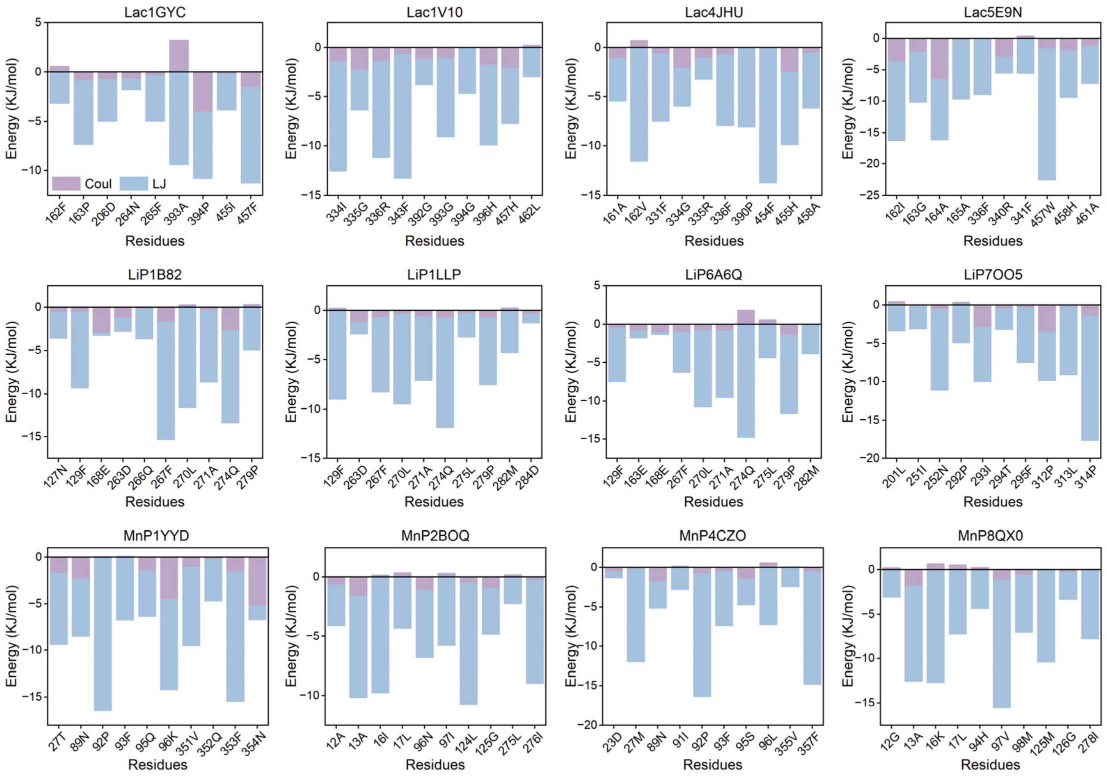

Figure S2. Interaction energy between substrate and lignin-degrading enzyme. Hydrophobic (blue) and electrostatic (purple) interaction energies are shown as bar plots.

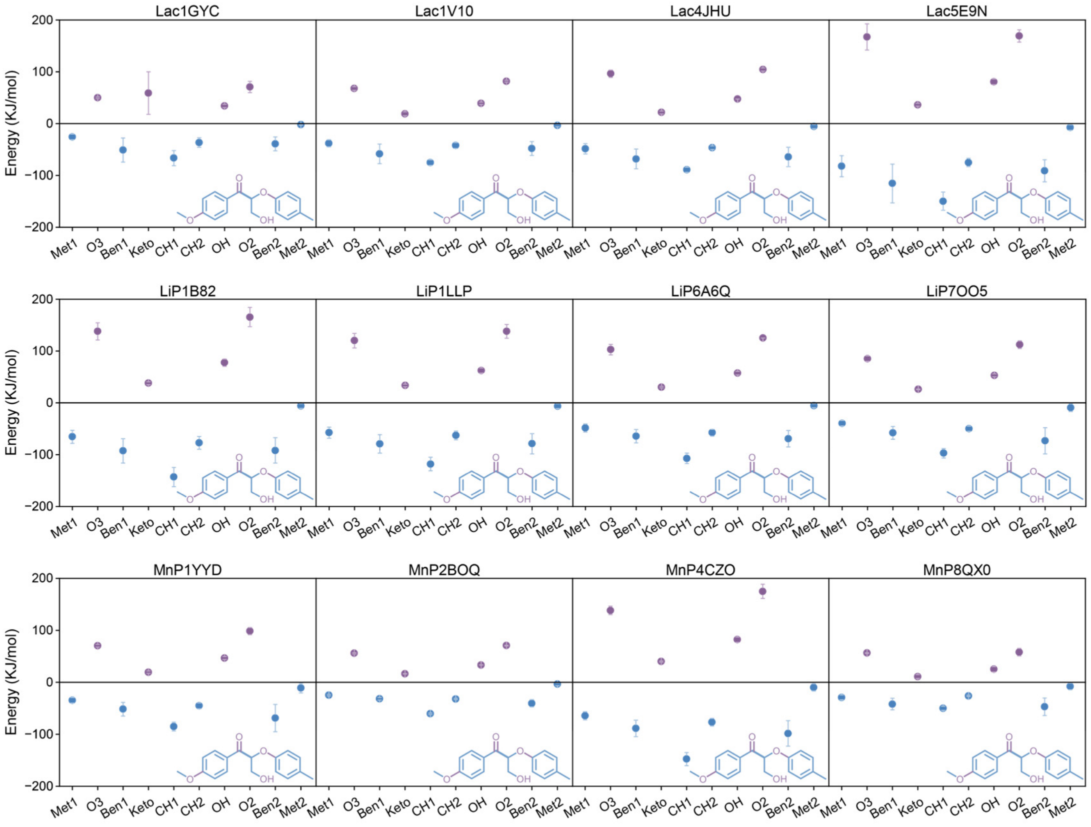

Figure S3. Binding Free energy between lignin-degrading enzymes and substrates. Bar colors indicate the nature of interaction: blue represents attractive forces, while purple represents repulsive forces, consistent with the coloring in the associated structural models.

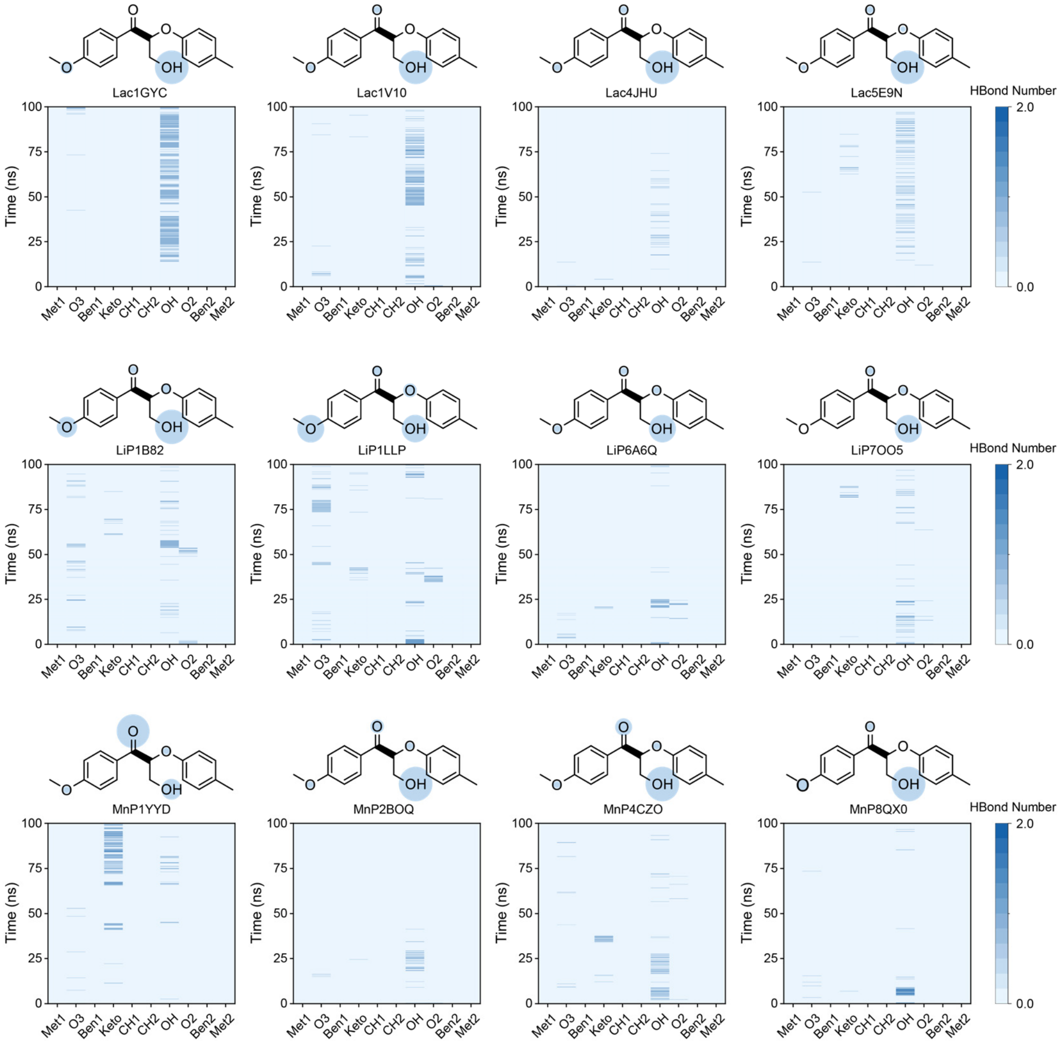

Figure S4. Hydrogen bonding interactions between substrate and lignin-degrading enzymes. Hydrogen bonds were analyzed from MD simulations. The size of the blue circles in the substrate's chemical structure indicates the strength of the hydrogen bonding interactions.
